# Boston Letter

**Published:** 1880-02

**Authors:** 


### Domestic Correspondence.

### ARTICLE X.

### BOSTON LETTER.

*Messrs Editors:*—In my last letter I made the remark that the question of the admission of women to the Massachusetts Medical Society, was not yet settled. I likewise ventured the opinion that if the matter were voted upon in a general meeting, the result would be the contrary of that reached in the October session of the councillors of the society. That a definite settlement is still in the future is proven by the fact that the question has already been raised as to whether the councillors have the power to bring upon the society such a radical change in its conditions without consulting the society at large. The censors are State officers, but are appointed by the councillors, and consequently may receive instruction from the latter. Nevertheless, although I have not been able to find it, I am told that there now exists in the by-laws of the society, a law passed in 1870, to the effect, that no law now exists which permits an interference with the duties and decisions of the censors. This being the case, some of the censors of Suffolk county are of opinion that the recent decision of the councillors relative to women, is an alteration, or at least a new reading of the by-laws. If it be an alteration, the councillors overstep their powers, because a change in the by-laws is illegal without vote of the whole society. If it be a new reading, then the by-law of 1870, to which I have alluded, renders the decision of the councillors to admit women, non-effective so far as the censors are concerned. For this reason the censors of this county doubt the legality of the recent action of the council and, consequently, have drawn up a protest to that effect. This protest has been communicated to the council and will be acted upon at their February meeting. While they thus remain in doubt as to their duty, the censors naturally are anxious for a proper solution of the question; for, believing they have no right to examine women, they cannot consistently do so. On the contrary, if they wrongly refuse to examine a qualified applicant, every man of them is liable to a fine of $400 for every such case.

Beside the law passed in 1870, and to which reference has been made, the censors of Suffolk county feel that the original by-law of the society in regard to the qualification of candidates has been misinterpreted. This by-law reads: "Every candidate for admission into the Massachusetts Medical Society must, by proper credentials and examination, satisfy the censors of said society that HE possesses the following qualifications for fellowship," etc. Now, the proper reading of this law depends upon the meaning given to the pronoun "*he*." This matter was made a subject of debate some years ago, at which time the opinion of Judge Hoar, in regard to the meaning of the pronoun was asked by the councillors. The Judge expressed the opinion that in its fullest sense "*he*" might mean *he* or *she* according to the judgment of the censors. This view clearly gives large margin, for, any body of censors may say: "If the spirit of the law means he or she, the letter means *he* and he only. So that in the minds of many members of the society the by-law is still considered debatable, and it will undoubtedly give rise to further discussion. If the censors of this county succeed in making a successful stand against the examination of women, these applicants would simply have to go to a county in which censors hold the opposite opinion and after a residence of a few months might apply for examination without hindrance. As you may see, the matter is in a muddle. At the coming meeting of the council the October vote may be reconsidered, and if change in the by-laws prove necessary, the whole question will be discussed in general meeting. We may then find that the majority will oppose the admission of women. That such will be the case is common opinion.

The authorities of Harvard University are making an effort to purchase the ground now occupied by the massive Cochituate reservoir, now unused, directly behind the State House, in order to erect thereupon a new building for the Harvard Medical school. This has given rise to criticism which culminated in a paper recently read before the Suffolk Historical Society, by a gentleman whose happiest mood in public, seems to be one of criticism, and who, I am sorry to say, does not love the Harvard Medical school as fondly as he should. In his paper, this gentleman calmly expressed the opinion that to expend $250,000 upon a new medical school building when the present one is good enough, would be simply nonsense; that the funds would be far better used in creating a new medical faculty, the present body of teachers being either effete reminiscences of other days, or the mere accidental appointees of a system of disgraceful wire-pulling. I need not say that less powder would have created an equally loud detonation. This paper was the expression of more minds than that of the writer, but, the reputation of the school is a reasonably good one, and as for wire-pulling, the less said touching a mere assertion the better. The teachers of the school are chosen for their ability. Those who know the truth and are able to be just, quietly smile and wonder whether it be possible that the critics find Harvard grapes too sour to be palatable. To offer one of them a professor's chair or a lectureship in the school which is the innocent object of their unmerited criticisms, would be an experiment whose result would pretty surely add new brightness to the jewel consistency and make doubly apparent the rarity of its virtues.

A most necessary and beneficial plan has just been brought into successful operation in Boston. I refer to a directory for nurses which has been opened at our Medical Library, and is under the immediate charge of the resident librarian, Dr. Brigham. The plan originated with Drs. F. C. Shattuck and C. P. Putnam, who, with three ladies of exerience, form the board of management. The system is simply this : At the library rooms is kept a record of the names, addresses, as well as present and future engagements of nurses who have graduated from training schools or who are recommended by physicians in good standing. Applications for nurses may be made at any hour of day or night, and the addresses of such nurses only as are known to be waiting for an engagement is given to the applicant. He has then only to proceed to her place of abode and secure her. Postal cards bearing the address of the library are furnished at cost to nurses, who are expected to keep the directory informed of their engagements. Failing to do this a nurse loses her place on the list and must register anew.

Each nurse upon registration pays a fee of fifty cents and responds to the following questions, the answers to which are written against her name: "How long have you been nursing? From what school having a diploma, if any? Is there any branch of nursing which you prefer to others? Do you wish to devote yourself exclusively to it? Are you willing to take meals in the kitchen if desired by employers? How much do you charge per day? Per week? To what physicians can you refer?" The replies to these queries give a résumé, as it were, of the nurse in question.

By circular, physicians are requested to recommend nurses to the directory, and likewise to refer to it all persons desiring nurses whether male or female, as well as manipulators and those who administer massage. Those who apply for nurses pay a fee of one dollar during the day, and two dollars from 8 p. m. to 8 a. m.

To the physicians whose names are given by nurses as references, the committee address circulars asking their opinions as to the desirability, capability and other qualifications of the nurses in question. Criticisms of any nature are likewise requested.

I have given you this much detail in order that you may see how perfect the system is. You will easily appreciate the relief which this directory brings to physicians, few of whom in the past have escaped the trial of being forced to drive about for hours, in order to find a nurse of *any* quality, much more a good one. Now, a dispatch or message is sent, day or night, to the directory, a good nurse is at once recommended, and the system goes like a clock. The nurse whose address is given is *always* found ready and waiting at any hour. This is a very excellent feature of the plan, which as a whole, acts with equal benefit in favor of physicians, laity and nurses. The comfort to be derived from an opportunity to read the history and gain a knowledge of the characteristics and antecedents of a nurse who is to work for a physician or serve a family, cannot be overestimated.

Moreover, the trustees of our city hospital have established a training school for nurses, and the school is conducted in such a careful, systematic and intelligent manner, that Boston will surely have an abundance of good nurses as well as a centralized means of obtaining them.

You have noticed the new form and dress of our *Medical and Surgical Journal.* Congratulations and compliments from all hands assure editors and publishers that the change is appreciated. The journal has developed rapidly within the past three years, owing to the liberality of the publishers and the active ability of Dr. G. C. Warren. The recent fire which destroyed the business house of the publishers, Messrs. Houghton, Osgood & Co., did not cause the suspension of the *Journal* even for one week.

We have our share of sickness. The summery-winterish weather has worked much harm in debilitating the health of the sensitive.

Boston, Jan. 13, 1880.

